# Prediction of CYP2D6 poor metabolizers by measurements of solanidine and metabolites—a study in 839 patients with known CYP2D6 genotype

**DOI:** 10.1007/s00228-023-03462-y

**Published:** 2023-02-20

**Authors:** Birgit M. Wollmann, Elisabet Størset, Marianne Kristiansen Kringen, Espen Molden, Robert L. Smith

**Affiliations:** 1 Center for Psychopharmacology, Diakonhjemmet Hospital, PO Box 23 Vinderen, 0319 Oslo, Norway; 2 Department of Life Science and Health, OsloMet – Oslo Metropolitan University, Oslo, Norway; 3 Department of Pharmacy, University of Oslo, Oslo, Norway; 4 NORMENT, Institute of Clinical Medicine, University of Oslo, Oslo, Norway

**Keywords:** CYP, Biomarkers, Phenotype, Metabolism, Precision medicine

## Abstract

**Purpose:**

Poor metabolizers (PMs) of the highly polymorphic enzyme CYP2D6 are usually at high risk of adverse effects during standard recommended dosing of CYP2D6-metabolized drugs. We studied if the metabolism of solanidine, a dietary compound found in potatoes, could serve as a biomarker predicting the CYP2D6 PM phenotype for precision dosing.

**Methods:**

The study included 839 CYP2D6-genotyped patients who were randomized by a 4:1 ratio into test or validation cohorts. Full-scan high-resolution mass spectrometry data files of previously analyzed serum samples were reprocessed for identification and quantification of solanidine and seven metabolites. Metabolite-to-solanidine ratios (MRs) of the various solanidine metabolites were calculated prior to performing receiver operator characteristic (ROC) and multiple linear regression analyses on the test cohort. The MR thresholds obtained from the ROC analyses were tested for the prediction of CYP2D6 PMs in the validation cohort.

**Results:**

In the test cohort, the M414-to-solanidine MR attained the highest sensitivity and specificity parameters from the ROC analyses (0.98 and 1.00) and highest explained variance from the linear models (*R*^2^ = 0.68). Below these thresholds, CYP2D6 PM predictions were tested in the validation cohort providing positive and negative predictive values of 100% for the MR of M414, while similar values for the other MRs ranged from 20.5 to 73.3% and 96.7 to 99.3%, respectively.

**Conclusion:**

The M414-to-solanidine MR is an excellent predictor of the CYP2D6 PM phenotype. By measuring solanidine and metabolites using liquid chromatography-mass spectrometry in patient serum samples, CYP2D6 PMs can easily be identified, hence facilitating the implementation of precision dosing in clinical practice.

**Supplementary Information:**

The online version contains supplementary material available at 10.1007/s00228-023-03462-y.

## Introduction

Cytochrome P450 2D6 (CYP2D6) is responsible for the metabolism of approximately 25% of all clinically used drugs [1]. Particularly, CYP2D6 plays a key role in the metabolism of psychiatric drugs, including many antidepressants and antipsychotics, but it is also of importance for antiarrhythmics, antiemetics, β-adrenoceptor antagonists (β-blockers), opioids, and anticancer drugs [1]. The *CYP2D6* gene is highly polymorphic leading to extensive interindividual variability of enzyme activity and systemic exposure of CYP2D6 drugs [2–4].

Based on *CYP2D6* genotype, patients are allocated to four different CYP2D6 phenotype subgroups, i.e., normal, intermediate, poor, and ultrarapid metabolizers. Among these most attention is paid to CYP2D6 poor metabolizers (PMs), who are carrying two lack-of-function alleles and therefore exhibit no CYP2D6 metabolism. Without appropriate dose adjustment, this often leads to several-fold higher exposure of drugs predominantly metabolized by CYP2D6 (e.g., risperidone, aripiprazole, atomoxetine, and metoprolol) and increased risk of dose-dependent adverse effects [2, 5, 6]. On the other side, suboptimal effects may occur among PMs when treated with drugs bioactivated by CYP2D6, such as the opioid prodrugs codeine and tramadol [7], as well as tamoxifen used for recurrent breast cancer [8].

Due to the major impact of genetic polymorphisms on the pharmacokinetic variability of CYP2D6 drugs, clinical use of genotyping as a tool for personalized dosing is increasing [9, 10]. However, the inability to capture non-genetic determinants of pharmacokinetic variability, e.g., concurrent use of interacting medication inhibiting CYP2D6, are limitations associated with the clinical use of pharmacogenetic testing on routine basis. Furthermore, most routine pharmacogenetic panels do not include rare variants encoding absent CYP2D6 metabolism. Thus, CYP2D6 phenotype biomarkers that can be easily and rapidly analyzed in blood samples for predicting individual dose requirements of CYP2D6 drugs may be favorable in tracing patients with deviate metabolism. From a safety point of view, the greatest clinical value would be to predict the PM phenotype to prevent serious adverse effects.

Recently, Magliocco et al. performed a non-targeted metabolomics study and reported that solanidine and its metabolites may have potential as CYP2D6 biomarkers [11]. Their evidence was based on the fact that plasma levels of solanidine metabolites decreased significantly during use of the potent CYP2D6 inhibitor paroxetine, as well as lower metabolite levels occurring in CYP2D6 poor versus normal-ultrarapid metabolizers. In this discovery study, the novel findings were based on a limited number of individuals and the predictive value of the various metabolite-to solanidine ratios were not compared.

In this study, we therefore aimed to compare the predictive value of solanidine and metabolite-to-solanidine ratios of seven solanidine metabolites as biomarkers for CYP2D6 in a large population of psychiatric patients with known CYP2D6 genotype.

## Methods

### Subjects

This retrospective study included CYP2D6-genotyped patients in which serum concentrations of psychiatric drugs had been measured during routine therapeutic drug monitoring (TDM) at Center for Psychopharmacology, Diakonhjemmet Hospital (Oslo, Norway) between March 2019 and April 2022. All the patients were Norwegian inhabitants confirmed by national identification numbers. Information about *CYP2D6* genotype was retrieved from the laboratory database, while existing high-resolution mass spectrometry (HRMS) data files of the respective patients were used for detection and semi-quantitative measurements of solanidine and its metabolites. Patients below 18 years or above 65 years were excluded. If multiple serum concentration measurements were available during the time span of the study, the last HRMS file was selected for solanidine metabolomics reprocessing. Patients with undetectable serum levels of solanidine *and* metabolite(s) were excluded from the respective statistical analyses. Information about potential use of the CYP2D6 inhibitors bupropion, fluoxetine, and paroxetine was retrieved during reprocessing of the HRMS data files.

The use of historical data in the present study was approved by the Regional Committee for Medical and Health Research Ethics and the Hospital Investigational Review Board.

### CYP2D6 genotype

The *CYP2D6* pharmacogenetic panel included the lack-of-function alleles (*Def*) CYP2D6*3 (rs35742686), CYP2D6*4 (rs3892097), CYP2D6*6 (rs5030655); the reduced-function (*Red*) variants CYP2D6*9 (rs5030656), CYP2D6*10 (rs1065852), and CYP2D6*41 (rs28371725), and copy number analysis to identify CYP2D6*5 (whole gene deletion; *Def*) and duplication of alleles (CYP2D6*1/Def/Red xN). Absence of *Red* or *Def* variants was interpreted as the fully functional, wild-type allele (CYP2D6*1). We divided patients into the following genotype-predicted CYP2D6 metabolizer subgroups according to the DPWG/CPIC consensus guidelines [12], i.e., poor metabolizers (PM; *CYP2D6Def/def*)*,* intermediate metabolizers (IM; *CYP2D6Def/red, CYP2D6Red/red* and CYP2D6*1/def), normal metabolizers (NM; CYP2D6*1/red, and CYP2D6*1/*1)*,* and ultrarapid metabolizers (UM: CYP2D6*1/*1 xN). A subset of patients was omitted from the main statistical analyses (i.e., gene ROC and linear regression analyses) but included in Fig. 2a for visual purposes, due to inconclusive genotyping (i.e., gene duplication in combination with reduced and/or non-functional variant alleles) since the genotyping assay did not determine which allele that was duplicated.

### LC-HRMS method for TDM of psychoactive drugs

The serum samples of which the study was based on had been analyzed on an ultra-high-performance liquid chromatography (UHPLC)-HRMS instrument, where a targeted multi-analyte method was applied for identification and quantification of 67 psychoactive drug analytes and metabolites for TDM purpose in clinical routine. Briefly, the serum samples were prepared by protein precipitation and the analytes were separated by an Xbridge BEH C18-coloum (2.6 µm, 2.1 × 75 mm; Waters, Milford, MA, USA) using gradient elution at 35 °C with a mix of ammonium acetate buffer (pH 4.8) and acetonitrile (20–52%). The LC system was a Vanquish UHPLC (Thermo Fisher Scientific, Waltham, MA, USA), consisting of a binary system of pumps, a column oven, a sample storing charger, and an autosampler. The QExactive mass spectrometer (Thermo Fisher Scientific, Waltham, MA, USA) were operated in positive ionization mode acquiring full-scan data at a resolution of 70,000 within the 100–1500 Da scan range. The compounds were quantified in full-scan acquisition mode, while accurate data-dependent MS2 (ddMS2) analysis was simultaneously triggered to permit confirmation of identification. The method is validated and used in routine analyses at Diakonhjemmet Hospital.

### Reprocessing of HRMS data files for solanidine metabolomics

For methods which acquire non-selective full-scan HRMS data, these data can be retrospectively reprocessed for compounds that were not targeted in the initial analysis, without the need of re-extraction and re-analysis of the sample. Solanidine and five solanidine metabolites (M412, M414, M416, M432, and M444), which were recently described by Magliocco et al. [11], were identified in the HRMS data files by accurate mass (the protonated molecular ion was present within a mass tolerance of 5 ppm) and isotope ratio. The MS/MS spectrums of the identified peaks, acquired using the full-scan ddMS2 mode, all showed a major fragment at *m/z* 98.0967, which further confirmed detection of the same solanidine metabolites as described previously [11]. The same MS/MS fragmentation pattern was also evident for two additional peaks, M402 and M440, which were discovered by use of the metabolomics software Compound Discoverer 3.2 (Thermo Fisher Scientific, Waltham, MA, USA). Molecular formulas, accurate *m/z*, average retention times and range in chromatographic peak areas for solanidine and the seven metabolites are presented in Supplementary Table S1. The identity of solanidine was confirmed using retention time and matched MS/MS spectrum by analyzing a reference standard purchased from Phytolab (Vestenbergsgreuth, Germany).

Initially, serum levels of solanidine and the seven metabolites were semi-quantified by retrospective reprocessing of stored TDM HRMS data files and subsequent extraction of the chromatographic peak areas (i.e., to be used for proper calculating the various solanidine metabolite-to solanidine metabolic ratios, MRs). TraceFinder 5.1 (Thermo Fisher Scientific, Waltham, MA, USA) was used for data processing. All peaks were integrated automatically, but all chromatograms were checked manually by a trained technician. Undetectable levels of solanidine and solanidine metabolites were truncated to half of the minimum value found for the corresponding analyte to enable proper calculations of metabolic ratios (i.e., solanidine, 1278 AUC; M402, 970 AUC; M412, 798 AUC; M414, 782 AUC; M416, 1117 AUC; M432, 844 AUC; M440, 963 AUC; M444, 629 AUC). Reprocessing of the HRMS data files was also used to detect use of the CYP2D6 inhibitors bupropion, fluoxetine, and paroxetine. Lastly, solanidine concentrations were also quantified using three calibrators in the range of 2–60 nmol/L prepared in solanidine-free serum for calculation of unadjusted or absolute solanidine concentrations.

### Patient randomization and statistical analyses

In order to accurately evaluate and validate the ability of solanidine and metabolites to predict CYP2D6-genotyped PMs, the study population, except patients using CYP2D6 inhibitors (only presented in Fig. 2b for visualizing purposes), was split into test and validation cohorts, in a 4:1 ratio, by stratified randomization of PMs and UMs. To determine potential statistical differences in demographics between the cohorts, Student’s *t*-tests and Fisher’s exact tests were used for comparisons of continuous variables and proportions, respectively. The present study’s outcome measures were the metabolite-to-solanidine metabolic ratios (MRs) and the unadjusted solanidine concentrations. The MRs of the various metabolites were calculated, and these ratios *and* unadjusted solanidine concentrations were ln-transformed prior to the statistical analyses to ensure normal distribution of the data.

To investigate the predictive value of the MRs and unadjusted solanidine concentration, the receiver operator characteristic (ROC) regression method was applied on the test cohort to determine optimal thresholds of the lnMRs and unadjusted ln-solanidine concentration with the highest sensitivity and specificity to predict CYP2D6-genotyped PMs. These thresholds were evaluated in the validation cohort using the following parameters: specificity, sensitivity, positive and negative predictive value (PPV and NPV, respectively), and Matthews correlation coefficient (MCC).

Furthermore, to obtain information on other potential factors impacting solanidine metabolism, multiple linear regression was used on the test cohort to define the impact of *CYP2D6* genotype, age and sex on the various lnMRs and unadjusted ln-solanidine concentration. The metabolite-to-solanidine ratio with highest explained variance *R*^2^ in the regression model was further investigated in relation to the various *CYP2D6* genotypes in the test cohort using ANOVA one-way with post hoc testing using Dunnett’s correction for multiple testing. Additionally, to assess the effect of CYP2D6 inhibitor use on solanidine metabolism, Student’s *t*-test was applied to compare the ln-transformed means of the MRs and unadjusted solanidine concentration in CYP2D6 inhibitor users that were CYP2D6*1/*1 carriers to the ln-transformed means in the CYP2D6*1/*1 subgroup in the test cohort.

All statistical analyses were performed in SPSS^®^, version 27.0 (IBM^®^ SPSS^®^ Statistics, Armonk, NY, USA). GraphPad version 9 (GraphPad Software, San Diego, CA) was used for graphical presentations. Alpha was set as 0.05, and the *P* values were Bonferroni-adjusted if not otherwise specified. The unstandardized B is given with standard error (SE).

## Results

Initially, 1014 patients were eligible for inclusion, in which 148 patients were excluded based on low or high age. The study population (*n* = 866) consisted of a male majority (58.9%) and the mean age was 41.3 years. The frequencies of the genotype-predicted CYP2D6 UM, NM, IM, and PM subgroups were 3.0% (*n* = 26), 53.5% (*n* = 463), 33.9% (*n* = 294), and 6.9% (*n* = 60), respectively. For 2.7% (*n* = 23) of the patients the CYP2D6 genotyping gave inconclusive results. Twenty-seven of the patients (3.1%) were using CYP2D6 inhibitors (*n* = 9 used bupropion, *n* = 16 used fluoxetine, *n* = 1 used paroxetine, and *n* = 1 used fluoxetine *and* paroxetine), and the distribution of the genotype-predicted CYP2D6 metabolizer subgroups among these patients were 14 NMs, 10 IMs, 2 PMs, and 1 inconclusive genotype. Solanidine was detected in 76% of the samples, while the metabolites M402, M412, M414, M416, M432, M440, and M444 were detected in 71%, 42%, 91%, 87%, 98%, 61%, and 71% of the samples, respectively. The various proportions of undetectable levels of solanidine and metabolites in relation to the various CYP2D6 genotype subgroups are given in Supplementary Table S2.

The population, excluding CYP2D6 inhibitor users, was split in a test cohort (*n* = 670 patients), which was included in the statistical analyses, while a cohort consisting of 169 patients (20%) was randomized to validate the predictive value of the optimal thresholds estimated from the test cohort. The characteristics of the population are summarized in Table 1. There were no significant statistical differences in cohort characteristics or proportions of undetectable levels of solanidine and/or the various metabolites, except for M444 (*P* = 0.012) between the two cohorts.

**Table 1:** Population characteristics

	Test cohort	Validation cohort	*P* value
Female/male	283/387	58/111	0.066
Age, y, mean (± SD)	40.8 (11.5)	41.8 (10.5)	0.324
CYP2D6 genotype:			
*1/*1 × N	21	5	> 0.99
*1/*1	283	65	0.384
*1/red	79	22	0.692
*1/def	180	51	0.387
Red/red	15	2	0.547
Def/red	29	7	> 0.99
Def/def (PM)	46	12	0.867
*1/red x N#	8	0	0.369
*1/def x N#	6	2	0.666
Red/def x N#	3	3	0.100
Solanidine conc., nM (SD)	1.73 (4.47)	1.67 (3.16)	0.866

Red, reduced function alleles: CYP2D6*9, CYP2D6*10 and CYP2D6*41

Def, lack-of-function variants: CYP2D6*3, CYP2D6*4, CYP2D6*6, and CYP2D6*5 (whole gene deletion)

*P* value from comparison of the test and validation cohort using Student's t-test and Fisher’s exact test

^#^ Not included in the receiver operator characteristic and multiple linear regression analyses. *PM* poor metabolizer

**Fig. 1:**
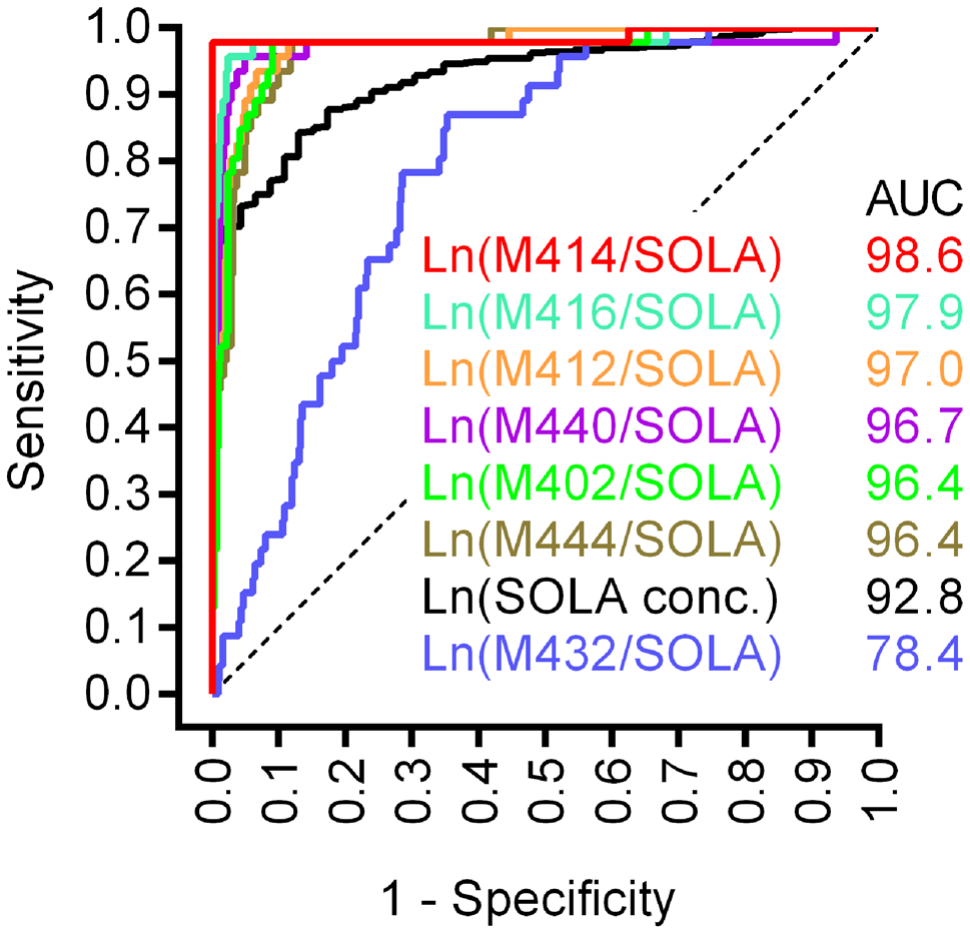
Receiver operating characteristic (ROC) curves used to evaluate the ability of the various metabolite-to-solanidine ratios and solanidine concentration to correctly predict a CYP2D6-genotyped poor metabolizer in the test cohort. Solanidine and metabolite levels determined from LC-HRMS analyses and genotype determined from CYP2D6 pharmacogenetic assays. Dashed lines represent the ROC curves for a random guess. SOLA, solanidine; conc, concentration; AUC, area-under-the-curve

In the test cohort, ROC curve analyses produced optimal threshold values with highest sensitivity and specificity for the prediction of CYP2D6 PMs based on the various MRs and solanidine concentration (Table 2). At these threshold values, sensitivity and specificity values ranged from 0.87 to 0.98 and from 0.65 to 1.00, respectively (Fig. 1), where the superior values represented the MR of M414.

**Table 2:** Evaluation of proposed threshold values to correctly predict CYP2D6-genotyped poor metabolizers in the test and validation cohort

	Test cohort					Validation cohort		
Variables	AUC_ROC_ (95%CI)	*P* value	Threshold value*	Specificity	Sensitivity	PPV (%)	NPV (%)	MCC
**M414/SOLA**	**0.99 (0.96, 1.0)**	** < 0.001**	** ≤ −4.28**	**1.00**	**0.98**	**100**	**100**	**1.00**
M416/SOLA	0.98 (0.95, 1.0)	< 0.001	≤ ** −**5.71	0.98	0.96	73.3	99.3	0.80
M440/SOLA	0.97 (0.93, 1.0)	< 0.001	≤ ** −**7.01	0.97	0.91	61.5	96.7	0.60
M402/SOLA	0.96 (0.94, 0.99)	< 0.001	≤ ** −**6.18	0.91	0.98	42.3	99.2	0.58
M412/SOLA	0.97 (0.95, 0.99)	< 0.001	≤ ** −**6.59	0.90	0.96	45.5	98.1	0.56
M444/SOLA	0.96 (0.93, 1.0)	< 0.001	≤ ** −**6.83	0.91	0.91	38.5	98.4	0.51
M432/SOLA	0.78 (0.73, 0.84)	< 0.001	≤ ** −**1.24	0.65	0.87	20.5	97.5	0.31
SOLA conc	0.93 (0.90, 0.96)	< 0.001	≥ 0.31	0.77	0.89	18.6	96.7	0.26

*PPV* positive predictive value, *NPV* negative predictive value, *MCC* Matthews correlation coefficient, *SOLA* solanidine, *AUC *area-under-the-curve, *conc. *concentration

^*^ln-values

The area under the ROC curves of all the lnMRs, and unadjusted ln-solanidine concentration was significantly higher from that under the y = x line (AUC = 0.5, *P* < 0.001; Table 2). When these threshold values were applied in the validation cohort, the lnMR of M414 had the maximum scores for both the positive and negative predictive value and the Matthews correlation coefficient (100%, 100%, and 1.00, respectively; Table 2). Evaluation parameters for the other metabolites and unadjusted solanidine concentration are shown in Table 2, and similarly to the lnMR of M414, the lnMR of these solanidine metabolites and ln-solanidine concentration had high negative predictive values (range 96.7–99.3), though lower positive predictive values (range 18.6–73.3) and Matthews correlation coefficients (range 0.26–0.80).

**Fig. 2:**
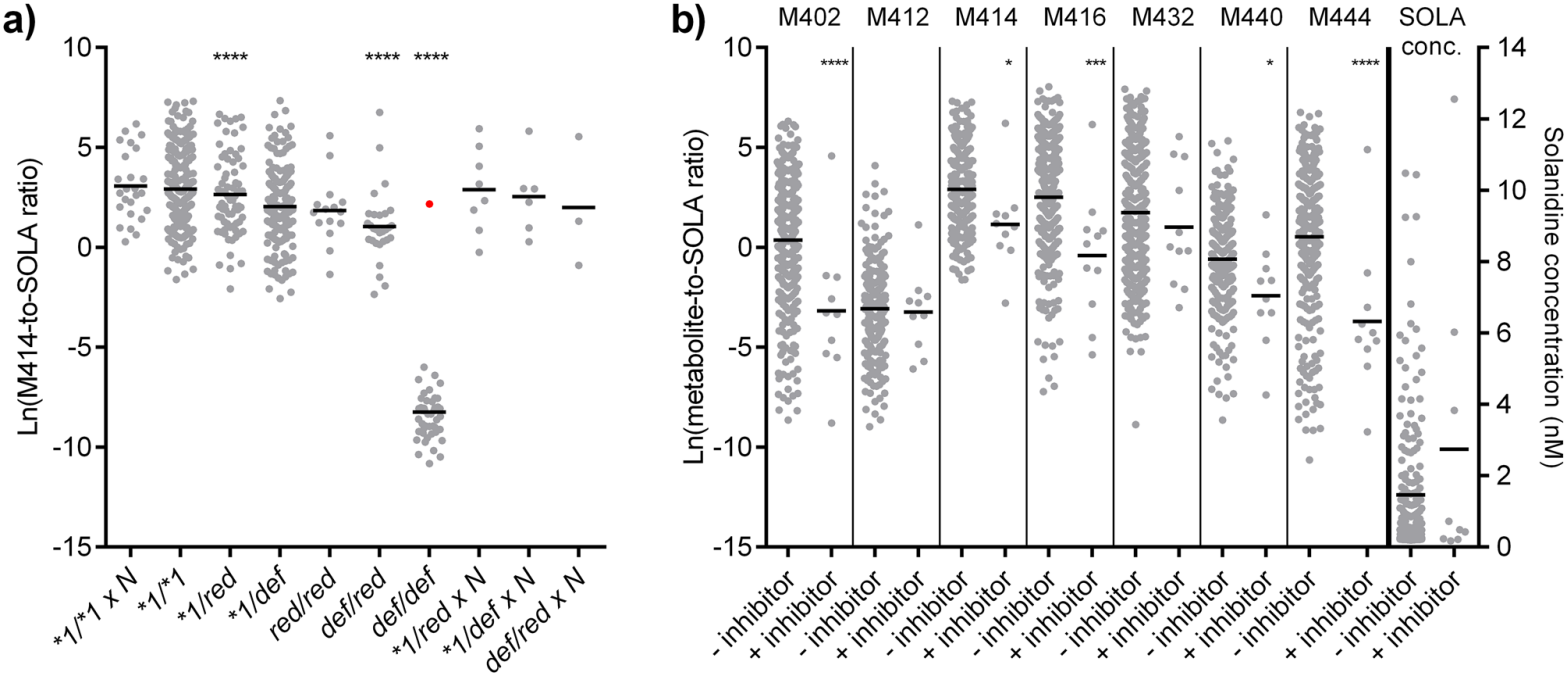
Effect of CYP2D6 genotype and CYP2D6 inhibitor use on metabolite-to-solanidine ratios and solanidine concentration. **a** Effect of CYP2D6 genotype on ln-transformed M414-to-solanidine metabolic ratios. Each dot represents a patient sample and the horizontal lines represent the means. Comparisons of the means were performed using ANOVA one-way with post hoc testing using Dunnett’s corrections for multiple testing with significance level of 0.05.**** *P* < 0.0001. The outlier (red dot) in the Def/def subgroup contained a rare *CYP2D6-2D7* hybrid gene which was misinterpreted as CYP2D6*4/*5 at the date of genotyping, but was reanalyzed and verified as CYP2D6*1/*4. **b** The graph shows the effects of CYP2D6 inhibitor use on the various metabolite-to-solanidine ratios and solanidine concentration in CYP2D6*1/*1 subjects. Each dot represents a patient sample and the horizontal lines represent the means. Comparisons of the means were performed using Student’s *t*-test. * *P* < 0.05, *** P < 0.001 and **** *P* < 0.0001. Red, reduced function alleles: CYP2D6*9, CYP2D6*10 and CYP2D6*41, Def, Lack-of-function variants: CYP2D6*3, CYP2D6*4, CYP2D6*6 and CYP2D6*5 (whole gene deletion), conc, concentration,

Multiple linear regression analyses were performed to estimate effects of CYP2D6 genotype, age and sex on the lnMR of the various solanidine metabolites, and ln-solanidine concentration (Table 3). Compared to the CYP2D6*1/*1 carriers, all the lnMRs were significantly lower in the CYP2D6 *1/def, *Def/red* and *Def/def* genotype subgroups, respectively (*P* < 0.05; Table 3). Correspondingly, mean unadjusted ln-solanidine concentrations were higher in the *1/def, *Def/red* and *Def/def* genotype subgroups, respectively, compared to CYP2D6*1/*1 carriers (*P* < 0.01; Table 3). Compared to CYP2D6*1/*1 carriers, the lnMR of all the solanidine metabolites were also statistically lower in the *Red/red* genotype subgroup (*P* < 0.05; Table 3), except the lnMR of M432 (*P* = 0.33; Table 3). The adjusted value of *R*^2^ from the linear models ranged from 0.12 to 0.68, where the MR of M414 had the highest explained variance and was further analyzed and illustrated (Table 3 and Fig. 2a). Age and sex did not significantly predict any of the outcome variables.

**Table 3:** The effect of CYP2D6 genotype, sex and age on metabolic ratios of various solanidine metabolites, and solanidine serum concentration. The effect of CYP2D6 genotype, age and sex on the ln transformed metabolic ratios, and solanidine concentration were estimated using multiple linear regression

	lnM402-to-SOLA ratio		lnM412-to-SOLA ratio		lnM414-to-SOLA ratio		lnM416-to-SOLA ratio		lnM432-to-SOLA ratio		lnM440-to-SOLA ratio		lnM444-to-SOLA ratio		ln-Solanidine conc. (nM)	
Variables:	B (SE)	P	B (SE)	P	B (SE)	P	B (SE)	P	B (SE)	P	B (SE)	P	B (SE)	P	B(SE)	P
n	601		505		634		636		650		533		611		653	
Intercept	0.51 (0.25)	0.042	−3.06 (0.21)	< 0.001	2.90 (0.15)	< 0.001	2.61 (0.22)	< 0.001	1.58 (0.24)	< 0.001	−0.49 (0.21)	0.021	0.80 (0.27)	0.003	−1.01 (0.10)	< 0.001
Male sex	−0.005 (0.26)	0.99	0.073 (0.21)	0.72	0.039 (0.16)	0.80	−0.006 (0.23)	0.98	0.22 (0.25)	0.38	−0.058 (0.21)	0.78	−0.05 (0.28)	0.86	−0.033 (0.11)	0.75
Age. Centered	0.005 (0.011)	0.67	0.008 (0.009)	0.38	0.001 (0.007)	0.84	0.011 (0.010)	0.26	0.007 (0.011)	0.53	0.003 (0.009)	0.78	0.013 (0.012)	0.26	0.0001 (0.005)	0.97
CYP2D6																
*1/*1xN	0.93 (0.73)	0.20	0.13 (0.64)	0.85	0.31 (0.46)	0.50	0.8 (0.65)	0.20	0.69 (0.72)	0.34	−0.050 (0.69)	0.94	0.94 (0.80)	0.24	−0.31 (0.30)	0.30
*1/red	−0.56 (0.42)	0.18	−0.20 (0.34)	0.56	−0.27 (0.25)	0.29	−0.38 (0.37)	0.30	−0.27 (0.40)	0.51	−0.23 (0.34)	0.49	−0.95 (0.44)	0.032	0.16 (0.17)	0.34
*1/def	−1.88 (0.31)	< 0.001	−071 (0.24)	0.004	−0.88 (0.19	< 0.001	1.58 (0.28)	< 0.001	−1.16 (0.30)	< 0.001	−1.50 (0.25)	<0.001	−2.32 (0.33)	< 0.001	0.39 (0.13)	0.002
Red/red	−3.87 (0.92)	< 0.001	−1.37 (0.57)	0.043	−1.06 (0.51)	0.038	−2.50 (0.81)	0.002	−0.83 (0.84)	0.33	−1.70 (0.72)	0.018	−3.85 (0.95)	< 0.001	0.57 (0.35)	0.11
Def/red	−3.44 (0.61)	< 0.001	−1.04 (0.46)	0.024	−1.87 (0.38)	< 0.001	−2.92 (0.56)	< 0.001	−2.04 (0.62)	0.001	−2.53 (0.50)	< 0.001	−4.76 (0.66)	< 0.001	0.93 (0.26)	< 0.001
Def/def	−8.75 (0.50)	< 0.001	−5.54 (0.37)	< 0.001	−11.2 (0.31)	< 0.001	−10.4 (0.45)	< 0.001	−4.16 (0.50)	< 0.001	−7.71 (0.39)	< 0.001	−9.38 (0.53)	< 0.001	2.78 (0.21)	< 0.001
Model R2	0.371		0.325		0.688		0.475		0.117		0.442		0.377		0.227	

The intercepts are calculated by the linear regression model and are the estimated mean ln-transformed metabolite-to-SOLA ratios, or solanidine serum concentration, in the reference populations, which consist of patients of female sex, of mean age and with the *1/*1 genotype. The unstandardized Bs express estimated increase/decrease in ln-transformed metabolite-to-SOLA ratios or solanidine concentration associated with a tested variable. The statistical analyses were performed at a significance level of α = 0.05, and compared to the *1/*1 subgroup, using Bonferroni-corrected *P* values. Red, reduced function alleles: CYP2D6*9, CYP2D6*10 and CYP2D6*41. Def, Lack-of-function variants: CYP2D6*3, CYP2D6*4, CYP2D6*6 and CYP2D6*5 (whole gene deletion), *SOLA* solanidine, *SE* standard error

Figure 2b shows the effects of CYP2D6 inhibitor use (*n* = 11) versus non-use on the lnMRs of the various metabolites and unadjusted solanidine concentration in CYP2D6*1/*1 subjects. The mean lnMRs of all the solanidine metabolites, except M412 and M432, were significantly lower in CYP2D6 inhibitor users compared to the CYP2D6*1/*1 subjects not using inhibitors (*P* < 0.05). There was no statistically significant difference in unadjusted mean ln-solanidine concentration compared with the non-inhibitor using CYP2D6*1/*1 subjects.

### Discussion

The present study in a large patient population shows that measures of solanidine metabolism could serve as biomarkers for predicting a CYP2D6 genotype encoding a PM phenotype. By applying the proposed threshold values determined from the test cohort to predict CYP2D6 PMs in the validation cohort, the MR of M414 exhibited maximum values for both the positive and negative predictive values and the Matthews correlation coefficient. This demonstrates that the M414-to-solanidine ratio can be implemented in clinical practice with a 100% accuracy in identifying patients with a genotype-predicted PM phenotype and be used as a biomarker for precision dosing of CYP2D6 drugs.

An issue related to the use of solanidine as a CYP2D6 biomarker is that some patients have undetectable levels of this dietary alkaloid substrate. The presence of solanidine in human serum for prolonged periods of time after potato consumption and in amounts dependent of potato consumption was reported by Harvey et al. in 1985 [13]. Potato intake is essential for the usability of solanidine as a CYP2D6 biomarker, and in the present study, we detected solanidine in 76% of the 866 serum samples by reprocessing of full-scan HRMS data files, which is similar to the detection frequency of 78% in 43 plasma samples measured by the more sensitive parallel reaction monitoring mode in the study of Magliocco et al. [11]. As potato intake habits of the included patients in the present study were unknown, the undetectable levels could be a result of both no intake or long time since intake, but also rapid solanidine metabolism. We therefore chose to truncate the undetectable levels in order to be able to calculate metabolic ratios, which cause more uncertainty to the ratios. Still, this study provides strong evidence that measures of solanidine metabolism could serve as biomarkers for predicting individual dose requirements of CYP2D6 substrates. Knowledge of CYP2D6 activity, particularly the PM phenotype is highly relevant clinical information as it could limit the increased risk of dose-dependent adverse drug events and therapeutic failure with regard to CYP2D6-dependent prodrugs in this patient population and also identify patients who are unlikely to respond on treatment with prodrugs bioactivated by CYP2D6 [14–16].

Despite increased implementation of *CYP2D6* genotyping in clinical routine and the commonly accepted use of genotype-predicted CYP2D6 phenotyping, phenotyping by measuring real-time enzyme activity is considered to be the golden standard method, as it reflects the combined effects of genetic, environmental, and endogenous factors on the metabolic phenotype [17]. The basic concept of phenotyping in vivo metabolic activity in patients is to administer a probe drug, where metabolism via the specific pathway to a great extent are mediated by the enzyme of interest. The major drawback of using probe drugs for phenotyping is, however, the invasiveness of the procedure, as well as the practicalities and time to get the final results on the patients’ phenotype. Thus, great efforts have been made to discover endogenous biomarkers reflecting in vivo CYP2D6 activity [18]. Solanidine is to our knowledge the first successful non-drug CYP2D6 biomarker, but should be defined as a dietary rather than endogenous biomarker.

Interestingly, one of the metabolites, M444, had been discovered previously in an untargeted metabolomic approach where it was assumed to be an unknown urinary endogenous metabolite and a product of a reaction catalyzed by CYP2D6 (named M1) [19]. During preparation of this manuscript, M444 was identified as 3,4-seco-solanidine-3,4-dioic acid by Behrle et al. [20]. The impact of the CYP2D6 PM phenotype on solanidine metabolism in the current population was very strong, and the fact that potato is a relatively common part of the Norwegian and European cuisine, biomarkers of solanidine metabolism have potential to act as a robust “dietary” biomarker for CYP2D6 activity that can be determined in most patients. The CYP2D6 biomarker potential of solanidine is further benefitted by its long half-life [13]; however, more studies are needed to assess the translational potential of our findings as it is expected that the potato intake habits would vary in different populations.

All the investigated MRs and unadjusted solanidine concentration proved useful to discriminate between individuals with a genotype-predicted PM phenotype and the other genotype subgroups. Generally, all threshold values resulted in high sensitivity; i.e., most genotype-predicted PMs were identified, but the precision was lower, which implicates potential misclassification of patients with other genotypes to the PM subgroup. However, the M414-to-solanidine threshold ratio demonstrated to be the best predictor and could identify 45 of 46 (98%) and 12 of 12 (100%) PMs in the test and validation cohort, respectively, without any misclassifications. In the test cohort, one of the patients in the PM subgroup had a remarkably higher lnM414-to-solanidine ratio than the other PM patients (shown in Fig. 2a as the one outlier (red dot) in the Def/def subgroup) and was reanalyzed. The latter revealed that this outlier contained a rare *CYP2D6-2D7* hybrid gene [21], which was misinterpreted as CYP2D6*4/*5 at the date of genotyping, but was verified as CYP2D6*1/*4. This genotype is more in line with the observed M414-to-solanidine ratio, and further demonstrates the ability of the M414-to-solanidine threshold ratio to correctly identify PMs. Recategorizing the outlier to the IM subgroup had minor effects on the overall results. The significance of CYP2D6 genotype on the metabolism of solanidine to M414 is also demonstrated by the highly explained variability from the multiple linear regression model.

As the present study had a retrospective design and did not include longitudinal measurements within patients, it was not possible to demonstrate changes in solanidine metabolism when patients are treated with CYP2D6 inhibitors. However, the lnMRs of all the metabolites, except M412 and M432, were significantly lower in CYP2D6 inhibitor users genotyped CYP2D6*1/*1 compared to CYP2D6*1/*1 subjects not using inhibitors. Even though our study population only included a small number of CYP2D6 inhibitor users, which again were treated with three different CYP2D6 inhibitors, our results show that the MRs are sensitive to CYP2D6 inhibition, although the MRs seem to be more sensitive to a genotype predicted non-functional CYP2D6 metabolism. Furthermore, these results coincide with the results from the before-and-after inhibition session (7 days of paroxetine intake) in the study by Magliocco et al. [11].

It should also be viewed as a strength that the threshold values are tested and validated in a large patient population and that reprocessing of the HRMS data files could detect comedication of the CYP2D6 inhibiting drugs bupropion, fluoxetine and paroxetine. However, the naturalistic nature of the current study is associated with some potential limitations. The retrospective study design entails that information regarding diagnosis, comorbidity and organ function are missing. Additionally, information regarding potato intake habits would have been useful. The translational potential of our results to other age groups also needs to be further investigated, as we only included an adult population (18–65 years) in the current study. Future studies that investigate the potential correlation with CYP2D6 substrates are required to further demonstrate the potential ability of solanidine metabolism to accurately predict CYP2D6 activity. The semi-quantification of the analytes based on reprocessing of HRMS data is related to some uncertainty, but the use of metabolic ratios of simultaneously detected solanidine and metabolite improves the robustness and may overcome some of the quantification variation of the method. Ideally, a quantitative method based on pure standards for all analytes is warranted.

In conclusion, the present study demonstrates that the M414-to-solanidine MR is an excellent predictor of the CYP2D6 PM phenotype. With access to patient serum samples, CYP2D6 phenotyping with solanidine can easily be performed by high-throughput, rapid, and cheap liquid chromatography-mass spectrometry analyses, hence facilitating the implementation of precision dosing of CYP2D6 drugs. These findings have clinical implications as individual dosing of CYP2D6 substrates can contribute to treatment optimization and prevention of adverse effects or predict lack of effect of prodrugs in CYP2D6-PM patients.

## Supplementary Information

Below is the link to the electronic supplementary material.

Supplementary file1 (DOCX 24 KB)

## Data Availability

The data that support the findings of this study are available from the corresponding author, BMW, upon reasonable request.
