# Supplementary material for: Prediction of CYP2D6 poor metabolizers by measurements of solanidine and metabolites—a study in 839 patients with known CYP2D6 genotype
Source: Eur J Clin Pharmacol. 2023 Feb 20;79(4):523–31. doi: 10.1007/s00228-023-03462-y (PMC10038974; doi:10.1007/s00228-023-03462-y)
Supplement: Supplementary file 1 — Supplementary file1 (DOCX 24 KB) [file 228_2023_3462_MOESM1_ESM.docx]

**SUPPLEMENTARY INFORMATION**

Prediction of CYP2D6 poor metabolizers by measurements of solanidine and metabolites – a study in 839 patients with known CYP2D6 genotype

*European Journal of Clinical Pharmacology*

Birgit M. Wollmann^1^, Elisabet Størset^1^, Marianne Kristiansen Kringen^1,2^, Espen Molden^1,3^, Robert L. Smith^1,4^

^1^Center for Psychopharmacology, Diakonhjemmet Hospital, Oslo, Norway

^2^Department of Life Science and Health, OsloMet – Oslo Metropolitan University, Oslo, Norway

^3^Department of Pharmacy, University of Oslo, Norway

^4^NORMENT, Institute of Clinical Medicine, University of Oslo, Norway

**Corresponding author:** Birgit M. Wollmann, Center for Psychopharmacology, Diakonhjemmet Hospital,

PO Box 85 Vinderen, 0319 Oslo, Norway

E-mail address: Birgitmalenetovik.wollmann@diakonsyk.no

Telephone: +47 22 45 46 57, Fax number: +47 22 45 46 98

**Supplementary Table S1**

Molecular formulas, accurate *m/z*, retention times and peak areas for solanidine and the seven solanidine associated metabolites

| Analyte | Molecular formula | Accurate [M+H]^+^ | Retention time (min) | Chromatographic peak area,  (range) |
| --- | --- | --- | --- | --- |
| Solanidine | C27H43NO | 398.3417 | 2.25 | 2 556 - 72 512 936 |
| M402 | C25H39NO3 | 402.3003 | 1.54 | 1 940 - 4 759 250 |
| M412 | C27H41NO2 | 412.3210 | 2.33 | 1 595 - 1 633 242 |
| M414 | C27H43NO2 | 414.3367 | 1.88 | 1 564 - 29 923 325 |
| M416 | C26H41NO3 | 416.3159 | 1.25 | 2 234 - 19 576 517 |
| M432 | C26H41NO4 | 432.3108 | 1.38 | 1 687 - 8 963 909 |
| M440 | C28H41NO3 | 440.3159 | 1.60 | 1 926 - 4 017 582 |
| M444 | C27H41NO4 | 444.3108 | 1.30 | 1 257 - 3 964 028 |

**Supplementary Table S2**

Proportion of undetectable metabolites and solanidine in samples with detection of either solanidine or metabolite.

|  | CYP2D6 genotype | | | | | | | | | |
| --- | --- | --- | --- | --- | --- | --- | --- | --- | --- | --- |
|  | *1/*1 x N | *1/*1 | *1/red | *1/def | Red/red | Def/red | Def/def | *1/red x N | *1/def x N | Red/def x N |
| **Test cohort** | N=21 | N=283 | N=79 | N=180 | N=15 | N=29 | N=46 | N=8 | N=6 | N=3 |
| M402 | 9.5 | 17.7 | 16.5 | 27.2 | 53.3 | 27.6 | 93.5 | 50 | 16.7 | 33.3 |
| M412 | 61.9 | 54.4 | 51.9 | 56.7 | 60 | 48.3 | 97.8 | 100 | 33.3 | 66.7 |
| M414 | 9.5 | 3.5 | 4.1 | 1.7 | 0 | 3.4 | 89.1 | 0 | 0 | 0 |
| M416 | 0 | 5.3 | 3.8 | 8.9 | 20 | 10.3 | 84.8 | 0 | 0 | 0 |
| M432 | 4.8 | 1.4 | 0 | 3.9 | 6.7 | 0 | 0 | 0 | 0 | 0 |
| M440 | 47.6 | 32.2 | 26.6 | 36.1 | 40 | 34.5 | 95.7 | 50 | 16.7 | 100 |
| M444 | 14.3 | 13.8 | 17.7 | 27.8 | 40 | 41.4 | 93.5 | 25 | 16.7 | 66.7 |
| SOLA | 42.9 | 32.9 | 26.6 | 18.3 | 20 | 13.3 | 0 | 50 | 16.7 | 33.3 |
| **Validation cohort** | N=5 | N=65 | N=22 | N=51 | N=2 | N=7 | N=12 | N=0 | N=2 | N=3 |
| M402 | 0 | 29.2 | 22.7 | 27.5 | 0 | 28.6 | 100 | NA | 50 | 100 |
| M412 | 80 | 55.4 | 63.6 | 54.9 | 0 | 14.3 | 100 | NA | 100 | 66.7 |
| M414 | 0 | 0 | 4.5 | 0 | 0 | 0 | 91.7 | NA | 0 | 0 |
| M416 | 0 | 6.2 | 13.6 | 7.8 | 0 | 14.3 | 83.3 | NA | 0 | 33.3 |
| M432 | 0 | 0 | 0 | 3.9 | 0 | 0 | 0 | NA | 0 | 0 |
| M440 | 20 | 30.8 | 45.5 | 39.2 | 0 | 14.3 | 100 | NA | 100 | 33.3 |
| M444 | 0 | 30.8 | 18.2 | 37.3 | 0 | 42.9 | 100 | NA | 50 | 33.3 |
| SOLA | 60 | 29.2 | 27.3 | 17.6 | 0 | 0 | 0 | NA | 100 | 0 |
| Red, reduced function alleles: *CYP2D6*9*, *CYP2D6*10* and *CYP2D6*41*.  Def, Lack-of-function variants: *CYP2D6*3*, *CYP2D6*4*, *CYP2D6*6* and *CYP2D6*5* (whole gene deletion)  SOLA, Solanidine | | | | | | | | | | |
